# Analectic

**Published:** 1829

**Authors:** 


### MISCELLANEOUS INTELLIGENCE

### I.—Analectic.

1. *On the Condition of the Blood and Blood-vessels in inflammation.*—Dr. Kaltenbrenner has recently published some interesting experiments which he instituted on these subjects. The successive changes which, by means of the microscope, he observed to take place in an organ at the moment of its being deprived of life, were the following:—On the approach of death, the column of blood in the arteries gradually diminishes in size, till, at last, the vessels contain only half of the usual quantity; the stream is uninterrupted, rapid, and without any visible pulsations, which, however, may be observed after some time, corresponding with those of the heart, and gradually becoming more and more distinct; at last, however, they become unequal and indistinct, and at the same time, the column of blood decreases, till it disappears entirely; the arteries are now quite empty, and organic life is extinct. Whilst the arterial stream is uninterrupted, no disturbance is observed in the veins; but as soon as the arterial circulation becomes unequal and irregular, the blood is accumulated in the veins; and from the moment that no more blood is carried into them, that which they contain stagnates entirely, retaining, however, for some time, an undulatory motion, passing into the branches, and then returning again; these undulations gradually diminish, and become reduced to smaller limits; the globules of the blood are conglomerated, all spontaneous motion ceases, and the mechanical laws determine its further direction. This undulation of the venous blood is observed, not only in dying animals, but also in parts divided from the living body, and in those which, by a very tight ligature, have been separated from the system. In these cases, the arteries are emptied as soon as they receive no more blood; the fluid of the capillary vessels, from this moment, is thrown into undulations, which press the blood towards the veins, and lastly, terminates in complete stagnation. This fact is a decisive proof, that the motion of the blood in the smaller arteries, and especially in the capillary system and veins, is, in some degree, independent of the action of the heart.

It is a general opinion, that after death the blood is equally distributed to all the organs of the body, unless any of them had been the seat of inflammation; this is not the case: in the extremities, the serous membranes, the lungs, &c. the blood retires from the capillary system into the larger veins, in other organs, as, for instance, in the spleen and liver, the capillary vessels do not completely empty themselves. It is very interesting to observe, that in fishes, the blood of the smaller vessels is not emptied into the veins, but that from the moment when the circulation is arrested, it is infiltrated into the cellular tissue, where it is found in reddish masses; a fact which can only be accounted for, by assuming that these small vessels are canals without proper parietes. In the liver of the frog, the same appears to take place, but not in that of the rabbit, which, after death, is found most beautifully injected.

In the spleen, the small vessels undergo a very singular change at the moment of death. During life, the distribution of the vessels in this organ is very similar to that in the substance of the liver; after death, the same phenomenon takes place as in the capillary system of fishes; the smaller arteries and veins, and the capillary vessels, emit their blood into the cellular tissue, where it is found in red masses; the larger arteries and veins only retain their blood, of which, in the smaller vessels, no trace can be discovered; this accounts for the general opinion that, in the spleen, the aterial blood is poured into cells, from which it is taken up by the veins; at the same time, it explains why all attempts to inject the arteries of the spleen from the veins have failed. Whoever examines the edges of the spleen of the mouse under a microscope, will be convinced that after death the blood of the capillary system is infiltrated into the parenchymatous tissue, but he will never, during life, observe its emission into cells.

On examining, after death, the mucous membrane of the small intestines, it appears even to the naked eye, that a small portion of the blood is retained in the capillary vessels, the rest being carried into the larger veins.

The changes which the circulation of inflamed parts undergoes after death, is very different from those observable in healthy organs. The blood is conveyed from all parts with accelerated motion, towards the centre of inflammation; the arterial is not changed into venous blood, and its coagulatory power is much increased. If in this state death takes place, the column of blood in the surrounding vessels diminishes in size, and the blood accumulates in the inflamed part, so that at last the peripheric vessels are perfectly emptied; at this moment the circulation ceases, but for a considerable time afterwards undulations are visible, by which the blood is gradually carried towards the centre of inflammation, and which insensibly terminate in stagnation. This motion, subsequent to the death of the animal, is also observed in the newly formed vessels. In a lesser degree of inflammation, the blood is only accelerated in its motion, and does not approach to a complete stasis; the centripetal undulations are also visible; but ultimately the blood is carried into the veins.

In such cases the inflamed parts exhibit hardly any redness after death.

It appears, that in some organs, inflammation is more disposed to form the inflammatory centres described above, than in others; in the latter division, to which the serous membranes seem especially to belong, exudation is most frequently observed. If cold water is injected into the peritoneal cavity, inflammation is soon excited, and quickly followed by exudation; the afflux of blood is so violent, as to make the membrane appear like a net work of injected vessels; from the moment that life ceases, the blood gradually leaves them, and is completely poured into the veins, so that, after death, but very slight traces of the preceding inflammation can be perceived.

The abdomen of an animal being opened, or its intestines and mesentery being drawn out, the contact of atmospheric air soon causes inflammation, which increases very rapidly in the mesentery, but slowly in the intestines. When, however, it has arrived at a certain pitch in the latter, it suddenly diminishes in the former, and gradually subsides, till at last its vessels are emptied, and the inflammation is confined to the intestines alone. The same phenomenon takes place if the mesentery is first irritated, and the intestine is afterwards exposed to any exciting cause. It seems, then, that inflammation is much more readily excited in the serous membranes, than in the organs which they envelope, but that it subsides very rapidly, and in the same proportion as it increases in the intestines. The tissue of the lungs appears also to be little disposed to form inflammatory centres, while in the liver the contrary obtains. The circulation of the latter organ is, even in the state of health, very slow and favorable to considerable accumulation of blood; in inflammation it is first accelerated, but gradually retarded, and lastly, a complete stagnation takes place. The same is observed in inflammation of the spleen.

Violent inflammation of the mucous intestinal membrane, often leaves no traces whatever; the blood with which, during life, the capillary vessels, were gorged, is, after death, so completely conveyed into the veins, as to render this membrane almost as pale as in its healthy state; this is even most striking in the most acute inflammation, so that, in this respect, the mucous are apparently very similar to the serous membranes.

When the capillary vessels are wounded, scarcely any extravasation appears to take place, only a few globules escape, and the circulation through the wounded vessels is not at all disturbed, but continues as before. If very small arteries are divided, the hæmorrhage is also very trifling; but the blood ceases to circulate through the wounded vessels, and passes entirely into the arterial branch next above the division. When a larger artery is divided, a considerable hæmorrhage ensues from the two ends, and the blood of the neighboring arteries is seen moving towards the wound as towards a centre; after some time, an undulatory motion is observed in the ends of the arteries; so that, at one moment, the blood moves towards the point of division, and, in the next, returns into the vessel; these undulations gradually decrease, till the movement of the blood, towards the divided extremities, ceases entirely, the blood being carried through the next arterial branches.—*Am. Jour. Med. Sci. No.* 6.

2. *On the Coagulation of the Blood.*—Dr. JOHN DAVY relates a number of interesting experiments on this subject in the *Edinburgh Medical and Surgical Journal*, for October, 1828. The following are his conclusions. 1*st*, The coagulation of the blood takes place independent of motion, and it is very little effected by motion, whether violent or moderate; 2*dly*, That it is not caused by the action of atmospheric air, or much affected by any kind of air that is not absorbable in water; 3*dly*, That it is not retarded by the introduction and absorption of carbonic acid gas; and *lastly*, That the action of reagents on the blood, or the fibrin of the blood, as regards coagulation, is exceedingly various, not to be anticipated *a priori*, and inexplicable on any one hypothesis hitherto advanced, the effect of each of them requiring special consideration, and further and minute experimental enquiry for its elucidation.—*Ib.*

3. *On the Therapeutic Properties of the Morphine.* By V. BALLY. —A little bitter taste in the mouth, is according to M. Bally, the only effect produced on the mouth and œsophagus by morphine. "It occasions no thirst, redness of the tongue or gums, or swelling of the tonsils. When given in moderate doses, morphine produces no loss of appetite or any other disorder of the digestive functions; wherein it differs greatly in its effects from belladonna. In most constitutions, however, it produces vomiting, if administered in full doses. This property it appears to possess in a very high degree, which is a great obstacle to its being used as a medicine. To avoid this effect, the dose at first should be very small, and should be very gradually and cautiously increased.

"The principal effect produced by morphine on the intestinal tube is constipation; hence it may be advantageously administered in cases of diarrhœa. But M. Bally has known several instances, where, after producing a constipated state of the bowels at first, a continuance of the medicine has brought on an abundant discharge of fecal matter. It sometimes produces colic pains about the region of the naval, but these are generally of short duration; and they cease of themselves, even when the medicine is continued, if the dose be not regularly increased. The author has some reason to consider the medicine as a vermifuge also, and he relates cases, where under its use, worms have been discharged by vomiting. It is not improbable, however, that if any other emetic substance had been administered in these cases, the same effect would have resulted. He has examined the intestinal canal in some instances in which morphine had been taken, but he could never discover any particular effects produced by it on the mucous membrane, probably owing to the smallness of the doses.

"With respect to the urinary organs, the action of morphine is very decided on the bladder. In almost every instance it produces a difficulty of passing the urine, and this amounts sometimes to a complete retention; but the dysuria generally ceases as soon as the medicine is omitted. This property of morphine, however, only manifests itself in men: M. Bally remarks that the remedy never produces the least difficulty of passing the urine in females. This is an extraordinary physiological fact. Morphine produces no sensible effect on the kidneys. The secretion of urine neither increases nor diminishes under its use, nor does the quality of the fluid become sensibly changed.

"M. Bally states in positive terms that the vascular system is by no means excited by the exhibition of morphine in moderate doses. He thinks that the reason which has induced some physicians to consider the remedy as an excitant has been from observing its effects where very large doses had been administered, and where the functions of the circulating system were disturbed in common with those of all the other organs. The author relates several cases in support of his opinion respecting this point; and in conclusion he states, that if the remedy have any effect at all on the heart and arteries, it is a sedative, not an exciting effect.

"In the next place we are informed that morphine has no tendency to produce hemorrhoids, that has no emmenagogue properties, that it will not provoke nasal hemorrhages, nor produce hemoptysis, that it will not allay cough in a satisfactory manner, that it is not diaphoretic, that it has no influence in the production of heat, that it will not oppress respiration, that it produces no flushing of the face or symptoms of asphyxia.

"The exhibition of morphine gives rise, in very many instances, to an intolerable itching of the skin. The irritation in some cases extends all over the surface, in others it is partial, confined more particularly to the nostrils, neck, loins, and the genital organs. The itching is not uncommonly accompanied by a cutaneous eruption.

"The brain and nervous system are the parts upon which morphine exerts its influence most particularly.

"Trembling and agitation of the muscular system are symptoms sometimes produced by the remedy, if continued for a length of time. It has also the property of occasioning dimness of sight, which renders it an improper remedy in amaurosis. M. Bally, having never administered morphine in very large doses, cannot speak with positiveness whether or not it have the property of occasioning dilatation of the pupils, but his opinion is that it has not. A young man took, in a mistake, a pill containing three grains of it; in this case no dilatation of the pupils took place. In cases of poisoning with opium, we have witnessed the pupils contracted to the apparent size of pins' heads. The author has noticed similar effects produced by the acetate of morphine. MM. Orfila, Magendie, Dupuy, and Bathelemy state that the pupils invariably dilate in experiments with morphine on animals. Respecting this fact, M. Bally observes that the iris of dogs, cats, and horses has a mobility much greater than that of man.

"Morphine appears to possess all the sedative effects of opium, and the action of both on the system is very similar. The former, however, is not liable to produce head-ache and the other symptoms of excitement which usually follow the exhibition of opium. The stimulating effects of opium have been generally attributed to the narcotine which enters into its composition; but some chemists are of opinion that the latter substance is nearly inert when deprived of morphine, and that the stimulating properties which it appears to possess when administered to animals, depend upon some portion of morphine remaining in combination with it. It is scarcely necessary to notice that a combination of these two substances may possess medicinal properties very different from those of either singly. Although pure narcotine may be inert in its effects on the system, still by its combination with morphine its latent properties will be developed, and will modify the therapeutic properties of the latter substance.

"Morphine and its salts have not yet found their way into general use amongst medical men. This is rather to be regretted, as their medicinal properties are, so far as observation has hitherto proved, better adapted than those of opium, for the purposes for which this is usually administered.

"In the production of sleep, and in some other effects, M. Bally says that there is no proportion between the therapeutic properties of opium and its extracts, and those of morphine. Fifteen grains of crude opium contains, on an average, one grain of morphine. According to this proportion, it might be expected that a given quantity of morphine would have fifteen times the effect on the system that the same quantity of opium would produce. This is however by no means the case. M. Bally observes that it may be admitted, as very probable, that a grain of the aqueous extract produces greater drowsiness, than a quarter of a grain of its salifiable base. This is a circumstance well worthy of attention.

"In summing up his observations on the action of morphine, M. Bally divides its effects into the direct and indirect. The former are nausea, vomiting, gastralgia, eructations, constipation, and intestinal pains: the latter are ischuria, itching, and all the cerebral symptoms. It is to be observed, however, that most of these symptoms occur only when the remedy is administered, in large or frequent doses. There is one very important advantage likely to result from the employment of the active principles of vegetable substances as therapeutic agents, namely, that they may be introduced into the system through the medium of the skin, in sufficient quantities to affect the constitution. Independently of the difficulty with which we sometimes meet of persuading individuals, particularly children, to take medicines, the stomach is often so irritable, as to reject every thing in the form, or under the name of medicine. M. Bally says that he has met with great success in administering the active principles of some remedies in this way, which he calls the *sub-epidermic* method. It consists in removing the epidermis by means of vesicatories, and in applying the active substances to the surface of the true skin. Of two persons affected with *chiroplegia*, or paralysis of the hands, the one was cured by the action of a grain and a half of strychnine, administered according to this method daily; the other had recovered the use of one hand entirely, and very nearly the entire use of the other when M. Bally wrote his memoir. Morphine in particular produces wonderful effects in rheumatism, lumbar neuralgia, and sciatica, when employed according to the sub-epidermic method. It always gives ease, as soon as it is brought in contact with the skin; and patients complain of great torments when its employment is discontinued for any time." *Memoires de l'Academie Roy. de Med.—Ibid.*

4. *On Strangury from Cantharides, and its relief.* By John Davy, M. D. &c.—"Strangury from cantharides, especially when applied to the skin for the purpose of blistering it, is of such frequent occurrence, and generally of such short duration, that it is commonly thought little of; and I hardly know which has received least attention from systematic writers—the explanation of the affection or its relief.

"To those who are conversant in hospital practice, and who apply themselves to pathological anatomy, it must be well known that this kind of strangury is connected with phlogosis of the lining membrane of some part of the urinary passages. I have observed it most frequently in the pelvis of the kidneys, and in the bladder of urine, and occasionally in the ureters, and in the upper part of the urethra. The part affected with inflammation is swollen and dark red, from blood extravasated into the cellular structure, under the epithelium. Sometimes, when the effect is most severe, blood, it is well known, is actually effused into the passages themselves, giving rise to bloody urine. Sometimes on the contrary, when the effect is slight, œdema, with very little redness of the mucous membrane is produced, unattended with strangury or any symptom indicating the specific action of the cantharides on the parts in question.

"During the prevalence of strangury, I need not observe the secretion of urine by the kidneys is either much diminished or almost totally suppressed:—At the same time, from the irritation of the bladder, a constant desire urges the patient to attempt micturition.

"The experienced practitioner can have little faith in the means commonly recommended for relieving this painful affection; such as the camphor mixture, *spiritus ætheris nitrici, &c.* Those who have tried these medicines most, if I may draw an inference from the result of my own observation, must place least confidence in their efficacy. The only means of relief which I have found almost constantly to succeed, is the introduction of the catheter, used not with the idea of drawing off urine, but for the purpose expressly in question. It should be employed with delicacy and caution, just slipped into the neck of the bladder, and kept in only a few seconds. The process is seldom very painful, and the relief is almost immediate.

"The rationale of the effect I shall not attempt to explain, as I have nothing but conjecture founded on analogy to offer on the subject."

*Edin. Med. and Sur. Jour. Oct.* 1828.—*Ibid.*

5. *On the tests by which Morphine may be made known in cases of Poisoning.* By V. Bally.—"The substance vomited by a dog which had been made to swallow twelve grains of acetate of morphine was a colourless fluid, without odour, slightly viscid. It turned frothy by agitation with solution of gum. It was about three ounces in quantity. Submitted to evaporation in a porcelain cup, it gave a small quantity of yellowish extract, of an odour of juice of meat, of a bitter taste, a little saltish, and it reddened tournesol paper. This extract, treated with boiling alcohol, separated into two portions, the one floculent, insoluble, formed of the mucous and gelatinous matter; the other, soluble in this liquid, was evaporated to dryness. The latter, redissolved in a little water, let fall floccules of greasy matter. Submitted to slow evaporation, the aqueous solution gave a deposit of prismatic crystals, of a yellow colour, which presented the followtng properties: they had a bitter taste; a solution of them in water precipitated, by the addition of ammonia, in white floccules Treated by concentrated sulphuric acid in a small glass tube, they disengaged a decided odour of acetic acid.

"Dissolved in weak nitric acid, these salts immediately gave a dark yellow solution, approaching to the colour of blood.

"This union of properties proved clearly that these crystals were acetate of morphine. The quantity obtained was about three grains.

"The stomach of a cat which had been poisoned by twelve grains of acetate of morphine was boiled for ten minutes in six ounces of distilled water. The filtered liquor was evaporated and treated with alcohol in the way already mentioned. The alcoholic solution was slightly yellow, and it furnished by evaporation an extract of a similar colour, only a little darker, of a saltish taste, followed by bitter, which manifested, by the addition of a few drops of nitric acid, a good yellow orange colour, approaching to red; phenomena which proved the existence of a small quantity of acetate of morphine.

"But it is remarkable that, in some instances, these reagents will not enable us to discover any trace of morphine in the stomach, intestines, heart, or in the blood taken from an artery a few minutes before the death of an animal which has been poisoned by this substance. A hound was poisoned by twelve grains, but no trace of it could be recognized in any of those parts. Two kittens died from the injection into the stomach of a solution of acetate of morphine, the one of five, the other of eight grains. The stomach of that which had received five grains showed unequivocal traces of the poison, easily distinguished by the reagency of nitric acid; whilst in the other nothing of the kind could be discovered. From these facts and several others of a similar nature, M. Bally concludes, 1st, that it is possible in many cases of poisoning, to discover, by chemical means, sensible traces of vegetable poison; 2d, that it is always in the viscera to which the poison is first applied that we are to look for its presence; 3d, that the matter thrown up by vomiting shortly after the injection of the poison into the stomach contains sensible quantities of it; 4th, that all the efforts made to discover it in the blood have been fruitless.—*London Med. and Surg. Journal, Oct. 1828, from the Memoires de l'Academie Royale de Medicine.*

6. *On the influence of Mercury on the functions of the Uterus.* By Alexander Colson, M. D. formerly House S urgeon of the Hopital des Veneriens, &c.—The functions of the uterus, both in a state of menstruation and impregnation, are often disturbed by strong medicinal agents; and as mercury is used in this country, to a greater extent than in any other, excepting, perhaps, America, it may be interesting to inquire, what are the effects of this medicine on the uterine functions? Such inquiry can best be prosecuted in a Lock-Hospital; and it is to this subject Dr. Colson has directed his attention.

The uterine derangements produced by mercury, he observes, are, mænorrhagia, amenorrhœa, and abortion. These effects Dr. C. illustrates by a series of cases, one or two in each class we shall briefly state.

1. *Mœnorrhagia.* A young woman, 18 years of age, was received into hospital on the 17th October, for the cure of vegetations about the labia. She had been regular in her menses for four years previously. She was put on the use of Van Sweiten's liquor, (solution of oxymuriate of mercury,) and eight days afterwards complained of pains in the abdomen, but especially in the loins and hypogastrium. The menses came on eight days in advance, and they continued to flow during more than two months that she remained in hospital. The mercury had no effect on the vegetations. They were excised—and in eight days afterwards, the patient was discharged cured. Four or five other cases are detailed, showing the mænorrhagic influence of mercury; but the above is a sufficient illustration.

2. *Amenorrhœa.* C. Virginie, aged 19 years, had contracted a syphilitic complaint, three months after she went on the town. She entered the Venereal Hospital, and was put on the use of the oxymuriate, which affected her mouth. Hitherto she had been regular in her courses; but now they stopped, and continued so all the time she was at the hospital. Two applications of leeches to the vulva were made in vain, as well as sinapisms, pediluvia, &c. She continued nearly three months in hospital, and left it in a state of amenorrhœa and general bad health, though the syphilitic complaint was cured. In about six weeks after she left the hospital, the menses reappeared. Six other cases of a similar kind, are detailed by the author.

3. *Abortion.* A female aged 24 years, six months pregnant, was received into the hospital, in the month of October, for the cure of gonorrhœa. She was subjected to *mercurial frictions!* At the end of a fortnight of this treatment, she complained, that she no longer felt the motions of the child, which previously were very distinct. She also complained of a sense of weight at the lower part of the abdomen, and in the groins. This state continued three or four days, and ended in the abortion of a fœtus dead, and apparently of about seven months.—Archives.

This case is followed by five others, where the premature expulsion of the fœtus was reasonably referable to the exhibition of mercury. The author remarks, that miscarriages are very common in the Venereal Hospital of Paris, though this accident is usually attributed to the syphilitic complaint, and not the mercury employed for its cure. Dr. C. is of a different opinion, and attributes the abortions to the mercury. We do not deem it necessary to enter into the reasonings adduced by our author, respecting the modus agendi of mercury on the fœtus. The facts are of more value than the arguments; and, as far as these facts go—and they are not very few in number—they offer fair testimony of the injurious effects of mercury on the functions of the uterus. How far the particular mode of administration in the Parisian Hospitals, may increase these bad effects of mercury, we are not able to determine, but this short notice of a lengthy memoir may convey some useful precautionary hints to the English practitioner.—*Med. Chir. Rev.—Ap.* 1829.

7. *Cataract and Glaucoma.*—There are now 23 cases of cataract attending at this hospital, (*the royal Westminster Ophthalmic,*) some lately operated on, others in more advanced stage of recovery, and a greater number either undergoing, or awaiting treatment or operation. In three cases the cataract is combined with glaucoma. Those students who have attended for even three months, have been able to point out the complication with the greatest ease. Mr. Guthrie has on several occasions lately described the difference between them. Where glaucoma existed he said,—"the disease usually came on about the middle period of life, from 45 to 55. In women, it generally followed the cessation of the catamenia, and was a point of great importance to be attended to at that period, inasmuch as most of the defects of sight which women laboured under about that period might be attributed to some negligence in their treatment at this time. Pain is usually felt in the head at intervals, which soon becomes fixed over the eyes, and on the side of the head, sometimes over one temple and eyebrow, sometimes on both; this is often excruciating, and with its increase the sight fails. At the end of from two to three years, the pains cease of themselves, even where no medical assistance has been sought; but not before the sight has been destroyed." It was very remarkable, Mr. Guthrie said, that Nature seemed to be satisfied with the mischief done, as soon as vision was lost; the pains then ceased, and the sufferer remained in comparative comfort, save that sight was gone. During this period, several slow, gradual changes took place in the internal structure of the eye, accompanied by corresponding external appearances. The cornea became very early less brilliant, the iris slightly discoloured, the sclerotica gradually changed from its natural white, to a yellowish, or even a shade of a leaden colour; tortuous vessels of a dark red colour slowly developed themselves at the circumference of the eye, spreading out their ramifications as they approached the cornea, which was frequently surrounded by them, the vessels seeming to penetrate, or in other words, to come out from within at a very little distance from it. Whilst these changes were going on, others of more importance were taking place within; the choroid coat was becoming varicose, the retina thicker and opaque, the iris irregular, the pupils fixed irregular, and dilated; vision daily decreasing. At last the lens becomes opaque, and of a greenish colour, is protruded against the iris, which is convex in appearance, and apparently changed in colour as well as in structure, and is pressed forwards against the cornea; or the pupil dilated irregularly to its utmost extent, leaves scarcely a distorted ring of iris visible at the edge of the partially obscured cornea through which it is seen. The eye-ball is harder to the touch. The patient has now lost all sense of light, but is free in general from pain. *Ibid.*

8. *On small doses of oil of turpentine for the cure of sciatica and other neuralgiæ.* By M. Martinet, M. D.—The employment of terebinthinates, in affections of the nerves, is of very ancient date; but it is only in modern times that the essential oil of terpentine has been fairly put to the test of experiment in the neuralgiæ, of which sciatica is one of the most obstinate and intractable. M Martinet has lately given an exposition of the results of his experience in 70 cases of sciatica, or other severe neuralgiæ of the extremities, where the oil of terpentine was employed, but in small doses—namely, about a fluid-drachm in the 24 hours, and taken at three different times. In this way, it seldom acted on the bowels; but generally produced a sense of heat in the stomach, and throughout the whole line of the intestinal canal, and occasional perspiration. A similar sensation was usually experienced in the nerve affected. The vehicle of exhibition was honey, syrup, or, what was the best of all, a mixture of syrup and calcined magnesia. The yolk of an egg is also a good ingredient in the vehicle. Where the digestive organs were very irritable, a few drops of laudanum were added. The modus agendi of turpentine in the cure of sciatica, or of any of the neuralgiæ, is quite unknown, and, therefore, we shall not waste time in discussing that point. The *resumee* of our author's experience with this remedy is as follow.

Of seventy patients who laboured under sciatica or other severe neuralgiæ of the extremities, fifty-eight were cured—namely, three by frictions, and fifty-five by the internal administration of the turpentine. Ten experienced only a more or less durable relief—and five derived no benefit whatever from the medicine. Two out of these last laboured under disease of the hip-joint, under which they ultimately sank. Of these 70 cases, 40 were acute, and 30 chronic. Of the 40 acute cases, 34 were cured—five were relieved—and one received no benefit. Of the 30 chronic cases, 24 were cured—two were relieved—and four remained without any mitigation of their complaints. Of the 58 patients who were completely cured, 34 were cured in less than six days—22 within twelve days—and three within six weeks. Of these 58 cures, 48 were cases of sciatica, two of which were cured by frictions. Three were crural neuralgiæ—four were brachial—and three facial. In the 10 cases which were only relieved (all of which were cases of sciatica) the treatment was suspended after the second day. In 21 cases, heat was felt along the tract of the nerve and in the limb affected; and 19 of these were completely cured. In 18 cases, heat was felt in the stomach and bowels. Three were affected with vomiting—but these had taken a larger dose than usual of the oil. Three had diarrhœa and colic. In five instances, the urine was augmented—four had dysury or strangury. In ten instances the perspiration was augmented. In one case, a degree of intoxication was produced—and in two others, a puritus was experienced over the body.—*Revue Med.*—*Ib.*

9. *On the form of the face and forehead; with a theory of their formation in different ages and nations.* By Dr. Milligan.—The figure of the face and forehead must, of course, depend upon that of the bones beneath, but the mode in which these are developed, and the relation which their developement bears to that of other bones of the body form the subject of Dr. Milligan's inquiry. Our ingenious author maintains that the evolution of the facial bones is governed by the following general laws:—

1. The external and internal plate of a flat bone are two different, distinct organs, merely connected with each other by diploe, as muscles are by cellular membrane, but each having an office corresponding to the organs contiguous to it.

2. Wherever those contiguous parts follow different laws of growth, the quicker growing bone separates from the slower, and leaves a space which is filled up by diploe, if no mucous membrane is near, and lined by mucous membrane, when that tissue is in the vicinity.

3. That this membrane presents the formation of diploe or cancelli, and, consequently, is the immediate cause of those sinuses that excavate the bones of the face.

4. Dr. Milligan proves, that the difference between the growth of the brain, which stops at the 7th year, and the growth of the face bones, which stops not till the 21st year, is in every case the cause of such separation, admission of mucous membrane, and formation of facial sinuses.

Hence nations or individuals that have the brain soon brought to the full growth, must have these facial sinuses large and the cheek bone wide, and the reverse. Hence, also, the large heads and small faces of Hydrocephalus are easily explained.

We subjoin the passage from the original work, which is now furnished with a copious alphabetical index, and some valuable plates and tables.

"The developement of the internal table of the skull, and, consequently, of the *frontal bone, follows the development of the brain; but the developement of the external table of the frontal bone follows the developement of the bones of the face.* Now, the brain we have seen, arrives at its full size in the seventh year; which, therefore is the period of completing the development of the internal table of the frontal bone. But the bones of the face continue growing to the twenty-first year; and hence it is, that anatomists find the dimensions of the frontal sinus go on increasing to that year; and the same authors generally find the sinus commence at the seventh year, because that is the time at which the nutritious arteries of the internal table cease to do more than support its vitality. Suppose that the day, or week, or month, after this has happened, a *line* in length and a *line* in breadth is about to be added to the external table by the arteries, in order to preserve its congruity with the transverse suture, which is still growing. It is manifest, that the arteries which make this new interposit must lay down their bony matter a line farther forward than before; while the absorbents, whose modelling action necessarily accompanies deposition, will remove that part which was in contact with the diploe from the now fixed vitreous table, in which no corresponding tendency to continue the union by diploe will be excited, even should points of diploe be thrown out by the still continued formative efforts of the vessels of the external table. Nay, these efforts soon must become abortive from another cause. Bichat has shown that the cells of diploe are without any membraneous lining—its plates are themselves bony membranes. When, therefore, the arteries of the anterior table advance its position forwards, to make it coincide with the nasal bones, an opening is offered behind, into which creep the vessels of the Schneiderian membrane which is in immediate contact at this point, irritated by the change of position, and at the same time equally rapid in their depositing action with the vessels of the external table. Hence, a membrane is rapidly shot into the nascent hollow, or *sinus*, which attaching itself to the outer aspect of the vitreous table, and to the inner aspect of the osseous table, forms an insurmountable obstacle to the rudest diploe that might join these two layers. For it is a mucous membrane, a class which scarcely ever forms adhesions, and is here almost a shut sac, whose sides are every day brought farther and farther asunder.

"I would rather say, that it is a similar penetration of this mucous membrane that forms the other cavities in the bones of the face, than that this cavity of the forehead is formed according to the model and for the same uses as those of the face, though both views are in some measure true. These cavities augment the sound of the voice, and, by rendering the bones hollow, equipoise the occipital prolongation so exactly upon its condyles that the weight of a small coin is often sufficient to turn the balance. To man alone are given posterior lobes of the brain, and he alone requires a projection of the anterior lobes, and corresponding facial bones, to balance the former—a state rendered doubly necessay by his erect position; but had those facial bones been solid, a useless weight of three or four pounds would have been superadded, producing a most injurious preponderance, and defeating the ends of nature.

"Thus, then, we have the true theory of the frontal sinus, and it becomes easy to explain what have hitherto been named anomalies."

*Ibid.*

10. *Pathology of Muscæ Volitantes.* By Dr. Parfait Landraw.—The following fact is so very curious, that had the statement not been verified by three or four respectable physicians and surgeons, we should have been inclined to view it as possessing the same kind of authenticity as that which is attached to Mr. Liston's lectures on aneurism, in the Lancet.

On the 17th August, M. Gally, physician of the perigneux hospital, brought to the author of the case, M. Audebert, an old magistrate, aged 70 years, who complained of having a derangement in the sight of his right eye, which gave him much inconvenience for some years past: namely, muscæ volitantes, black specks, and other images assuming various forms. On examination, the pupils were found to be rather contracted; and, when Dr. P. looked attentively into the posterior chamber of the right eye, Dr. P. could perceive numerous small bodies floating about in the posterior chamber of the eye, glittering with a kind of phosphoric brilliancy. The Doctor at first distrusted the evidence of his own senses, and proposed a dilatation of the pupils, by means of belladonna, which was effected, and then M. Gally and himself, together with M. Renaud, surgeon to the hospital, and Dr. Vidal, had a full opportunity of clearly observing the corpuscles above mentioned floating about in the posterior chamber of the eye, when the eye was in motion, and settling down, so as almost entirely to disappear, when the organ was quiescent. These phenomena could be distinguished by the naked eye; but, to remove ambiguity, a lens of some power was employed, and the same facts were verified.

We need hardly remark, that muscæ volitantes have been hitherto accounted for in a manner very different from the above. They have been attributed to a particular condition of the internal membranes of the eye to varicose vessels expanded on these membranes, or in the humours of the eye—and to an affection of the retina. Demours, however, has observed that these muscæ volitantes arise and subside, according as the eye is in motion or at rest—a fact which accords with the phenomena that appeared in the present case.

There is one difficulty in supposing that these bodies are located in the vitreous humour of the eye—namely, the cellular structure of that humour. Our author endeavors to get over this difficulty, by supposing that the cellular texture of the humour is broken down before the muscæ volitantes take place. There is no doubt that the vitreous humour is occasionally found so fluid as to escape in operations on the eye. The case is very curious, and should attract the attention of oculists and others to the subject. The occurrence of muscæ volitantes is very common, and, therefore, opportunities for observation will not be rare.—*Revue Med.—Ibid.*

11. *Tartar on the Teeth.*—Many hypotheses have been published respecting the nature and source of the earthy material which accumulates on the teeth, termed tartar.

Professor Berzelius, in a work on animal chemistry, says, when it first settles on the teeth it is mere hardened mucous, and that during its decomposition, phosphate of lime is produced, which adheres firmly to the enamel. M. Serres says that it is secreted by minute distinct glands, and not a deposit from the saliva. Professor Hertz, in his popular treatise, considers it a consolidated morbid secretion of relaxed or irritated gums; and many dentists attribute it to decomposition of animal and vegetable food lodged between the teeth. Mr. La Beaume has lately ascertained, by microscopical examination, that this collection is produced in the same manner as coral, by animalculæ resembling the *medreposa oculata.* By means of a solar microscope of strong magnifying power, we have seen them in a very lively state; and, from the cellular organization of the tartar, we have no doubt of the correctness of Mr. La Beaume's theory. The same has been observed by Mr. Cooper, a scientific chemist and geologist of London. Mr. La Beaume is decidedly of opinion that, after the tartar, which, like coral, is a mere nidus, adheres firmly to the teeth, the animalculæ burrow into the teeth, and, by insinuating themselves between the teeth and gum, occasion disease in both; but the secretion from them is often so offensive as to contaminate the breath. Mr. La Beaume has made numerous experiments with different mineral, vegetable, and animal acid, and with alcohol, to ascertain their effects on the animalculæ and on their habitation, and it is a curious fact, that of all the articles he has employed, the true *vinegar* acid, (not the pyroligneous acid, which is now generally sold for it,) almost instantaneously killed the animalculæ, and acted powerfully in decomposing the concretions, so that they were easily removed by a brush. The more powerful acids, in the same state of dilution, and alcohol, seemed to have little effect on the animalculæ. In order to destroy the animalculæ and their eggs, and to decompose the production which protects them, Mr. La Beaume recommends the teeth to be brushed every morning with the *vinegar* acid, (acidum aceticum *verum*,) diluted with rose water, and immediately afterwards to make use of the levigated areca nut charcoal, as recommended by Professor Hertz, in his popular Treatise on the Management of the Teeth.

The use of the diluted acetic acid every morning will, in the course of a few days, entirely remove the tartar, and the regular employment of the areca charcoal and tincture of rhatany every, or every other morning, will effectually prevent the generation of the animalculæ.—*Bost. Med. & Surg. Jour.*

12. *New mode of administering Quinine.*—Four cases of facial neuralgia, which resisted the ordinary treatment, yielded to the administration of one grain of powdered quinine, in two grains of snuff, mixed and used as common snuff. This dose was always sufficient, and in from two to three days the patients were cured, as if by enchantment. The cases occurred to Dr. Richet, of Metz, and are related in his thesis presented to the faculty of medicine of Strasbourg.—*Ibid.*

13. *On the supposed Existence of Active Molecules in Mineral Substances.* By Robert Bakewell, Esq.—In our last number, p. 200, we published a notice of the supposed discovery, by Mr. Brown, of active molecules in mineral substances We felt well convinced at the time that there had been some error, being aware of the numerous ones to which microscopical researches are liable, and we are now happy to point out some interesting observations and experiments by Mr. Bakewell, which disprove this imagined discovery, and show some of the circumstances from which it probably originated. "I have made," says Mr. B. "repeated observations on several mineral substances, which Mr. Brown says are chiefly composed of these active molecules; and though in some instances I was at first persuaded that I had seen the motions of the molecules similar to those of the smallest species of Infusoria, a more careful examination proved that I was mistaken, and that the motions were derived from causes that had not been properly appreciated. In these experiments it is absolutely requisite to employ fresh distilled or fresh boiled water. The Thames water and water in cisterns generally contain numerous animalcules. I chiefly made use of single lenses from one-twelfth to one twenty-eighth of an inch focal length, varying in magnifying power, from 100 to 220

times in linear dimensions: the use of the compound microscope is, I think, inadmissible in such delicate observations. With the lowest of the above-mentioned powers, a particle less than one-twenty-thousandth part of an inch in diameter is distinctly perceptible, and the form of a particle of twice that diameter may be observed. To make use of higher powers than what are absolutely required, renders the examinations more difficult, and the result more uncertain.

"To obtain glass in a highly comminuted state, I took the powder-blue used by laundresses, which I still further triturated. The small portion of the oxide of cobalt which enters into the composition of this glass, could not be supposed to paralyse the action of the molecules, as all the metals that can be reduced to powder and are said to contain these active molecules.

"By making use of powder-blue, I had the advantage of seeing when all the larger particles were deposited. Among other substances which I more particularly examined, were finely powdered adhesive slate, mountain cork, quartz, flint, and kaolin, from a specimen of the best kind used in the manufacture at Sevres, given me by M. A. Brogniart. When a drop of water containing any of these substances was placed under the microscope, I perceived particles in motion, which continued for some time, and then was scarcely discernible, but on laying my hand upon the table the motion recommenced, and was evidently produced by a current in the drop; although many particles appeared to be more influenced by it than others, which occasioned a change in their relative positions. Hence I became convinced, that in order to make the experiment properly, the microscope should be placed on a support not liable to be effected by vibrations of any kind, and I therefore placed the instrument on a support made for a telescope stand, so constructed as to prevent vibration when examining the more delicate double stars. I found that even the pulsation of my body occasioned an oscillatory motion of the particles, when the microscope was placed upon a table. After repeated trials, I became satisfied that whatever motions may appear to take place among the particles, for some time after the drop of water is first placed under the microscope, they will soon subside, if not kept up by agitation from external causes. In London, as an excellent practical philosopher, the late William Nicholson justly observed, it is scarcely possible to avoid the effects of vibration; this may be seen by the continual tremors visible on the surface of mercury placed in a basin. Now, if the particles of dust that fall on the surface of the mercury could be brought under a powerful microscope, they would appear in constant motion. Let us suppose the mercury to be changed for water, a similar effect will take place, and the particles that may sink under the surface will represent the particles of dust in a single drop; and Mr. Brown informs us that the whole of the London dust is composed of active molecules. I am fully convinced, however, that their activity in a drop of water, as well as when dancing in the sunbeam, is derived from external agitation. The very force of gravity constantly drawing the particles downwards must not be overlooked, for it is not contended that the vitality of inorganic particles is sufficient to keep them permanently suspended in water. The observer's breath, and the constant evaporation of the drop, have also a tendency to produce counter-currents. A drop of water placed under the microscope, may be regarded as equal, in apparent magnitude, to a quart of the same fluid in a water-glass, and will be subject to the various currents that may be produced by agitation in the larger quantity; but the slightest movement which occasions a displacement of the particles, even the thousandth part of an inch will, under a high magnifying power, make them appear to perform a long voyage. Some of the animalcules discovered by Lœwenhoeck, the motions of which excited so much surprise, he informs us, never travelled farther than a hair's breadth.

"It is highly improbable that we shall ever be able to reduce mineral substances to their ultimate molecules by pulverization. Their essential qualities remain the same after pounding as before; and, could we construct microscopes that would magnify twenty thousand times in linear dimensions, we should see in pounded quartz, flint, &c. fragments and pebbles the size of walnuts, exactly resembling those of the same minerals at the foot of a mountain, and it is difficult to believe that they would gain active moving powers by simple immersion in water. Still the philosophical world is greatly indebted to Mr. Brown, for having directed the attention of naturalists to this curious subject. About ten years ago, I was informed that Mr. Bywater, an ingenious optician, now residing in Liverpool, had discovered moving animalcules in coal-ashes, pounded marble, and other mineral substances. Little interest was then excited by the supposed discovery; it required an eminent naturalist, like Mr. Brown, whose merits are well known and highly appreciated in his own country, and on the continent, to direct public attention to statements so much at variance with our preconceived notions of matter. If, contrary to my expectation, after all due caution in the observations, it should be finally established that mineral substances are composed of active molecules, what new views of nature will the discovery unfold! Beds of siliceous sand, like those on our Hampstead heath, are only awaiting a further process of trituration, to be awakened into life by the torrent that shall bear them into the ocean; and the geologist, while he contemplates the organic remains of a former world embedded in solid rocks, must regard the rocks themselves as the parents of future living beings. But who shall presume to say that we have at present discovered all the properties which the Creator has communicated to material substances? It should be borne in mind, that less than a century since, latent heat, electric and galvanic energy, and crystalline polarity were unknown as important agents in nature; and that philosophers attempted to explain the phenomena of thunder-storms, and even of vital action, on mechanical principles. It will not be denied that many important processes take place in the mineral kingdom, which cannot now be explained by the agency of known causes, but await the discovery of other principles for their satisfactory elucidation.

"As it is probable that many persons may be desirous of entering this new field of inquiry, it will materially assist them in forming an accurate judgment of what they observe, to provide pepper water, and other vegetable or animal infusions, that they may from time to time compare the motions of the real animalcula infusoria with those of the supposed active molecules; and, if modern philosophy did not disdain to profit by the illustrations which common life frequently offers, I would recommend them to look attentively at the bubbles, or the crumbs that float on the surface of a basin of tea, and they will soon be convinced, that change of relative position is not a sufficient proof of spontaneous motion or vitality."—*Mag. of Nat. His. March*, 1829.—*Am. Jour. Aug.* 1829.

14. *On the Physiological Effects of Oxygen Gas upon the Animal System.* By S. D. Broughton, Esq.—Although it has long been known that the respiration of pure oxygen gas is destructive to life, some differences of opinion have existed with respect to the physiological conditions of the animals subjected to its influence; and also with regard to the quantity of oxygen consumed under these circumstances, compared with that consumed by the respiration of atmospheric air. With a view to elucidate some of these points, Mr. B. confined rabbits, guinea-pigs, and sparrows, in glass jars, inverted over water containing oxygen gas, obtained from black oxide of magnese by a red heat. The animals at first appeared to suffer no inconvenience from the respiration of the gas; but after some time, (generally in about an hour,) their breathing became hurried, and their circulation accelerated. This state of excitement was followed by an opposite one of debility; the respirations became feeble, and were more slowly performed; loss of sensibility and of the power of voluntary motion gradually supervened, till the only remaining visible action was a slight one of the diaphragm, occurring at distant intervals On opening the body under these circumstances, and also after the entire cessation of the movements of the diaphragm, the breast was found to be still in vigorous action; the blood in every part of the vascular system, both venous and arterial, was of a bright scarlet hue; it was remarkably thin, and rapidly coagulated; and the temperature of the body continued undiminished. If, before the diaphragm has ceased to act, the animal is removed from the vessel to the open air, it generally either recovers spontaneously, or its animation may be restored by artificially inflating the lungs with atmospheric air. Mr. B. found that the gas in which animals had thus been confined till they died, retains its power of rekindling a blown out taper, and of sustaining for a time the life of another animal introduced into it; and he hence deduces the inference that it does not contain so great an excess of carbonic acid as the gas left when animals have perished by confinement in atmospheric air. He considers the train of symptoms induced by the respiration of pure oxygen gas as analogous to those which follow the absorption of certain poisons into the system.—*Lon. Med. Gaz. May.* 1829.—*Ibid.*

15. *Silver found in the viscera of a person who had used the Nitrate of Silver.*—Dr. Wedemeyer, of Hanover, has published in *Rust's Repertorium*, the case of an individual who used the nitrate of silver internally, for the cure of epilepsy; the disease was removed, and the skin assumed a blue colour. The patient was afterwards affected with disease of the liver and dropsy, of which he died. On post mortem examination, all the internal viscera were found more or less stained of a blue colour, similar to that of the external surface. The plexus choroides and pancreas were submitted to chemical examination by M. Brande, and a portion of metallic silver obtained.—*Ibid.*

16. *Rhubarb Plant.*—"The plant that yields the fine rhubarb of commerce, having been long involved in obscurity, is now discovered to be the *Rheum australe;* it flowered in June last, in the collection of A. B. Lambert, Esq. at Boyton House, Wilts. The stem of the cultivated plant is from seven to ten feet high; the leaves are cordate, ample, numerous, and of a grass green colour; the flowers are smaller than in any of its congeries, and of a dark, or blood red colour; the seeds are dark red, with a highly polished surface; when bruised, they emit a powerful odour of rhubarb. It is perfectly hardy, and ripens its seed readily in this climate.

"Dr. Wallich, of the Calcutta Botanic Garden, sent over some dried specimens of the true rhubarb plant, and some seeds, from which Mr. Lambert raised a number of plants. We were informed by Mr. Anderson, of the Apothecaries Garden, at Chelsea, that he had proved the stalks of this species to be powerfully purgative, when employed in the same manner as those of the *Rheum undulatum*, in pies.

"It seems probable, that in a rich, loamy soil, we shall be able to cultivate this valuable article of the materia medica advantageously. For a fuller account, and a coloured figure of the plant, see *Sweet's British Flower Garden*, October, 1828."—*Lon. Med. and Sur. Jour. Jan.* 1829.—*Ibid.*

17. *On the Treatment of Intermittent Fevers by the Sulphate of Quinine, applied to a denuded surface of the Skin.* By Professor C. Speranza,—In a great number of cases it is impossible to administer certain remedies, either from the difficulty of swallowing or the vomiting they produce, the insurmountable disgust which they inspire, or the youth of the patient; and in some instances it is probable that the liquids of the stomach modify the remedies introduced into this organ. Under these circumstances, the employment of medicines by the endermique method offers a very valuable resource. Professor Speranza has employed in this manner the sulphate of quinine, in the treatment of a considerable number of cases of intermittent fever. He relates, among others, five cases of tertian fever, which yielded immediately to the application of the sulphate of quinine, applied upon a surface deprived of the epidermis by a vesicatory. In all the patients the fever had existed for many days, without manifest local alteration, except two, which presented gastric symptoms. Without premising purgatives, a blister was applied at once, and in most of the cases, even the day of the fever: the sulphate of quinine was applied at the end of the access, or the commencement of the apyrexia. The arm was chosen to apply the blister to, as the most convenient for dressing: the skin was rubbed well with strong vinegar, to hasten the formation of the bulla produced by the blister; immediately after the epidermis was removed eight or ten grains of the sulphate of quinine incorporated in a small quantity of pomatum was placed on the denuded surface. By employing this method, the fever has most frequently ceased after the first application, without its being necessary to make a second. Not only fevers whose primitive type were tertian, but those which had originally been continued and become intermittent, were treated in this way with success. In no case did a relapse take place, which is so common after the administration of quinine internally. In some subjects in whom symptoms of organic disease necessitated the use of evacuants or topical applications after the disappearance of the fever, this last did not reappear during the course of the secondary treatment. The application of the blister during the febrile paroxysm did not produce any disorder of the urinary organs or irritation of the neck of the bladder.—*Archives Generales de Medecine, from the Annali Universali di Med. Nov. Dec.* 1828.—*Ibid.*

17. *Ergot of Rye in Chronic Uterine Discharges.*—Dr. Marshall Hall is of opinion that the ergot possesses considerable powers in controlling chronic uterine discharges. In a very severe case of menorrhagia, alternating with leucorrhœa, of four years duration, the ergot was given in doses of five grains four times a day, beginning just before the expected appearance of the catamenia, with the effect of retarding the catamenia and suppressing the leucorrhœa. In several cases of leucorrhœa he has used the ergot with decided success. The effects of the remedy are usually perceived at the end of five days; it is recommended, however, to persevere in its use for a somewhat longer period.—*Lon. Med. and Phys. Jour. May,* 1829.—*Ibid.*

19. *Taliacotian Operation performed with success.*—Since the supplemental noses which the poet tells us, "Taliacotius from the brawny part of porter's b—m first cut," many such useful appendages to the human face divine, have since been fashioned by the barber-surgeons, and surgeons not barbers, who have followed them. The following, we are told, was moulded and applied rather for use than for show.

Ann Nicholson, æt. 37, was admitted on the 16th Aug. 1827, with no part of the nose remaining, except, the right ala and the columna. All had been swept away, leaving an unsightly chasm, bounded at the sides by the nasal processes of the superior maxillary bone, and above by the os frontis. As the edges of the opening were covered, in most parts only by a red shining cuticle, which the slightest injury caused again to ulcerate, it was thought that, by giving them a soft covering from the neighboring parts, a recurrence of these ulcerations might not only be prevented, but the hideous deformity in some degree remedied.

"The case was by no means a promising one, but the patient being willing, and a consultation having sanctioned it, the operation was performed in the following manner: the right ala and columna being retained, and the edges of the chasm being pared all round, a flap, two and a half inches in length, and one and a half in breadth, having somewhat the shape of a jibsail, was taken from the left side of the forehead, the integuments over the centre having been destroyed by previous ulcerations, and now occupied by linear cicatrices. The flap was left attached at its lower and narrower part, near the inner angle of the orbit; a half turn was given to it at this point; it was then laid down and fitted to the raw edges of the opening, and retained *in situ* by four stitches. A piece of India rubber was introduced by the new-made nostril, and passed upwards so as to raise the flap in imitation of the bridge of the nose. Soft lint was laid along the raw edges, and over all a light compress. The wound of the forehead was approximated, as much as possible, by adhesive straps. The dressings were removed on the fourth day when the flap was found in perfect opposition, and united throughout most of its extent. Thereafter it was dressed daily, and in about three weeks had quite cicatrized, remedying very materially the deformity, and leaving a soft cushion over the sharp edges of the nasal processes of the superior maxillary bones. The cicatrix on the forehead became of very small dimensions—a mere line. She continued in the house till 6th November when she was discharged, in good health, and greatly amended in appearance. It should be mentioned, that the communicating part of the flap never was divided, the prominence formed by the twist serving to remedy the deformity arising from the want of the bones of the nose."—*Med. Chir. Rev. Ap.* 1829.

20. *Fungous Tumours from the Gum and Alveoli.*—Most surgeons have had opportunities of witnessing the hard, immoveable, red, and, for the most part, exceedingly vascular, tumours which grow from the gum, generally from that of the upper jaw. The structure of these tumours is frequently exceedingly like scirrhus, at least it is semicartilaginous, and, in one or two instances, we have found on the section a portion of sabulous matter of bone. Three cases of the kind are related by Dr. Maclachlan. In the first, the tumour occupied the situation of the two middle incisors of the lower jaw, and had originated two years previously, in consequence of breaking one of them. The disease was removed by the knife, but the profuse hæmorrhage which ensued, required the repeated application of the actual cautery, together with compression, by means of a firm roll of lint, and a bandage passed across the angles of the mouth and round the chin. The wound healed kindly, and no appearance of reproduction of the disease has taken place.

In the second instance the tumour which was firmer, more nodulated, and paler in colour, was of twenty years' standing, had already been twice removed, and grew from the upper jaw. It was extirpated by the knife, and the actual cautery afterwards applied. The third was one of a similar history and structure, apparently rather a production of the bone, than of the covering soft parts, removed by Dr. Anderson from the posterior part of the palate.

We believe it is now very generally admitted, that the mere excision of this tumour is not sufficient to ensure a cure. The actual cautery should be afterwards freely applied to the bone from which it grows, or, under particular circumstances, the saw. With all these precautions, the evil is not always completely destroyed.—*Ib.*

21. *Small-Pox.*—Dr. George Gregory presents us, in a very interesting paper in the *London Medical Gazette*, for February, 1829, with the experience of the Small-pox Hospital, during the year 1828.

One hundred and ninety-three patients were admitted with small-pox, or some of its modifications, during the year. Of these seventy-one had undergone regular vaccination, leaving a cognizable and for the most part good cicatrix. Of the remaining one hundred and twenty-two who were unprotected, fifteen had been "cut for the cow-pox ineffectually," and one hundred and seven took the disorder without having submitted to any kind of preparatory process. None were admitted subsequent to variolous inoculation The total number of deaths was sixty-six, rather more than thirty-three per cent. Of the one hundred and twenty-two unprotected cases, sixty-three died and fifty-nine recovered, being a mortality of above fifty-one per cent. Of the seventy-one who had undergone vaccination, three only died, and sixty-eight recovered, being a mortality of only a little more than four per cent.

The few deaths that have occurred among those who suffered under small-pox after undergoing vaccination, may lead some to suppose that the fatality of small-pox has diminished. Such, however, is not the fact. In 1794 the rate of mortality in the hospital was thirty-five per cent.; in 1795, thirty-one per cent.—average of the two years, thirty-one. In 1828, as we have already stated, with seventy-one admissions subsequent to vaccination, the rate of mortality was no less than thirty-three per cent. and if we consider only those who were unprotected by vaccination, fifty-one per cent.

But it is not only lessening the mortality that vaccination has shown its beneficial influence, it has also shortened the amount of suffering. The duration of each patient in the hospital shows pretty correctly the severity of the complaint, for, with few exceptions, they were all admitted on the second, or, at the farthest, on the third day of the eruption; nor were they discharged until perfectly restored to health. Thirty-seven patients having small-pox after vaccination, were discharged cured, having been in the hospital less than twelve days. Eleven of these were less than eight days in hospital. Forty-seven out of sixty-eight were discharged cured within a fortnight from admission. Eight only remained in the hospital upwards of three weeks. On comparing this with the experience of the hospital prior to the days of vaccination, it appears, that in 1794, out of two hundred and thirteen patients three only were discharged within a fortnight from the date of admission, viz. one on the fourteenth, and two on the thirteenth day; and in 1795, out of one hundred and eighty patients two only were so discharged, viz. one on the tenth and one on the twelfth day after admission. By far the larger proportion of the discharged cases remained upwards of three weeks and many several months.

Dr. Gregory endeavors to explain the fact of the average rate of mortality by small-pox not having diminished since the discovery of vaccination, by supposing that the *modified* cases have taken the place of the *natural distinct*, and he says, "it will be found that the latter, formerly so common, is now become a very rare form of the disorder, insomuch that, for the last two months, not one case bearing that character has been admitted into hospital."

"We may generalise," he adds, "one step further, and lay it down as a great general law, that there are certain constitutions which are naturally *patient* under the influence of the variolous poison. Prior to the discovery of vaccination, these must of course have been far more numerous than at present. In consequence of the disuse of inoculation, and the fewer foci of the poison now to be met with, the disease fastens itself at present chiefly upon those whose constitutions are predisposed by weakness, or some less cognizable circumstance—whose systems are exceedingly irritable under the operation of the poison, and who therefore suffer severely from it. Hence the greater number of confluent and semi-confluent cases in the practice of the present day among the unprotected part of the community; while the proportion of mild and recoverable cases is kept up by *vaccination*.

"If I have reasoned rightly on the phenomena before me, the following may be looked upon as a correct statement of the influence of vaccination upon the human body. Those persons whose constitutions are naturally patient under the influence of the variolous poison, and who, prior to the discovery of vaccination, would have had the mild or distinct kind of small-pox, are by vaccination rendered perfectly secure from the attacks of small-pox. Those again whose constitutions are naturally *irritable* under the variolous poison, and who would formerly have suffered severely from it, even by inoculation, are now, by vaccination, rendered in a great degree secure from its mortal effects. The few who do fall victims to small-pox after vaccination, are those whose constitutions are so peculiarly susceptible and irritable as to bid defiance to any kind of medical treatment, whether prior to, or during the attack."—*Amer. Jour. Med. Sci. Aug.* 1829.

22. *Mercury detected in Swaim's Panacea, by Chemical Analysis.* By R. Hare, M. D. Professor of Chemistry in the University of Philadelphia. (Communicated in a letter to Dr. Hays.)—In February, 1827, I procured from the shop of Mr. Peter Lehman, druggist, sign of the Golden Lion, High street, a bottle of Swaim's Panacea, for the purpose of testing it for certain active metallic preparations which it was supposed to contain. Upon examination I found it to be clogged with so large a proportion of syrupy matter, that I considered it an object to get rid of this matter before prosecuting my examination. I therefore diluted about two-thirds of a bottle of the panacea with about two gallons of water, and added some yeast in order to induce fermentation.

Other subjects having absorbed my attention subsequently, the liquid remained covered up in the glass vessel into which I had introduced it, until nearly a year had elapsed. I then transferred the whole of the liquor, then much attenuated by fermentation, and the matter which had subsided from it, into a flat stoneware vessel, and placed it in my evaporating oven. From this situation the vessel was not removed, until the contents had been converted into a dry, blackish, porous crust. Of this crust the greater part was subsequently removed from the evaporating vessel, and being rolled up in paper was placed upon a shelf. Towards the close of the last summer, I happened to examine the crust attentively, when I observed on it some globules of metalic mercury. On further examination with the aid of a lens, I discovered it to be so replete with mercurial globules, that whenever any fresh portions of the crust were opened by means of a knife, more of them were observable. The crust was subsequently shown to Dr. Physick, Dr. Horner, and other intelligent friends, and it has been preserved in a bottle. I should have communicated these results to the public sooner, had I not been in hopes to have repeated the examination by another process; but not having as yet found it convenient to realize that intention, and as you deem it of importance that the facts which I have mentioned should be published, I send this statement to you for the American Journal.—*Ibid.*

23. *Some account of the Siamese boys, lately brought to Boston.* DEAR SIR,—In compliance with your request, as well as in obedience to what I consider to be a professional duty, I undertake to give some account of the Siamese Boys, and particularly of the medium, by which they are united together.

The boys are supposed to be about 18 years old. They are of moderate stature; though not as tall as boys of that age in this country. They have the Chinese complexion and physiognomy. The forehead is more elevated and less broad than that of the Chinese, owing to malformation. They much resemble each other; yet not so much but that upon a little observation, various points of dissimilarity may be noticed.

The substance by which they are connected, is a mass two inches long at its upper edge, and about five at the lower. Its breadth from above downwards may be four inches, and its thickness in a horizontal direction two inches. Of course it is not a rounded cord, but thicker in the perpendicular, than in the horizontal direction. At its lower edge is perceived a single umbilicus, through which passed a single umbilical cord to nourish both children in the fœtal state. Placing my hand on this substance, which I will denominate the cord, I was surprised to find it extremely hard. On further examination this hardness was found to exist at the upper part of the cord only, and to be prolonged into the breast of each boy. Tracing it upwards, I found it to be constituted by a prolongation of the *ensiform cartilage of the sternum*, or extremity of the breast bone. The breadth of this cartilage is an inch and a half; its thickness may be about the eighth of an inch. The cartilages proceeding from each sternum meet at an angle, and then seem to be connected by ligament, so as to form a joint. This joint has a motion upwards and downwards, and also a lateral motion, the latter operating in such way, that when the boys turn in either direction, the edges of the cartilage are found to open and shut. The lower face of this cartilage is concave, and under it is felt a rounded cord, which may be remains of the umbilical cord. Besides this there is nothing remarkable felt in the connecting substance. I could distinguish no pulsating vessel.

The whole of this cord is covered by the skin. It is remarkably strong, and has no great sensibility, for they allow themselves to be pulled by a rope fastened to it, without exhibiting uneasiness. On shipboard, one of them sometimes climbed on the capstan of the vessel, the other following as well as he could, without complaining.

When I first visited the boys, I expected to see them pull on this cord in different directions, as their attention was attracted by different objects. I soon perceived that this did not happen. The slightest impulse of one to move in any direction, is immediately followed by the other; so that they would appear to be influenced by the same wish. The harmony in their movements is not the result of a volition, excited at the same moment. It is a habit, formed by necessity. At an early period of life it is probable they sometime differed. At present this is so rarely the case, that the gentlemen who brought them, has noticed only a single instance. Having been accustomed to use the cold bath, one of them wished it, when the weather was cool; to which the other objected. They were soon reconciled by the interference of the commander of the ship. They never hold a consultation as to their movements. In truth, I have never seen them speak to each other, they converse constantly with a Siamese lad, who is their companion. They always face in one direction; standing nearly side by side, and are not able, without inconvenience, to face in the opposite direction, so that one is always at the right, the other at the left. Although not placed exactly in a parallel line, they are able to run and leap with surprising activity. On one occasion, a gentleman, in sport, pursued them round the ship, when they came suddenly to the hatchway, which had been inadvertently left open. The least check would have thrown them down the hatchway, and probably killed one or both; but they leapt over it without difficuly.

They are quite cheerful; appear intelligent; attending to whatever is presented to them, and readily acknowledging any civility. As a proof of their intelligence, it is stated, that in a few days, they learned to play at drafts well enough to become antagonists of those who had long been versed in the game

The connexion between these boys might present an opportunity for some interesting observations in regard to physiology and pathology. There is, no doubt, a net work of blood vessels and some minute nerves passing from one to the other. How far these parts are capable of transmitting the action of medicines and of diseases, and especially of what particular medicines and diseases, are points well worthy of investigation. Captain Coffin informed me they had never taken medicine since they had been under his care. Once they were ill from eating too heartily, but were relieved by the efforts of nature. He thinks that any indisposition of one extends to the other; that they are inclined to sleep at the same time; eat about the same quantity, and perform other acts with great similarity. Both he and Mr. Hunter, the gentleman who united with him in bringing them here, are of opinion, that touching one of them when they are asleep, awakens both.

The pulsations of the heart are exactly alike in both boys. I counted seventy-three pulsations in a minute, while they were sitting; counting first in one boy, then in the other. I then placed my fingers on an arm of each boy, and found the pulsations take place exactly together. One of them stooping suddenly to look at my watch, his pulse became much quicker than that of the other; but after he had returned to his former posture, in about a quarter of a minute, his pulse was precisely like that of the other boy. This happened repeatedly. Their respiration are of consequence, exactly simultaneous.

This harmony of action in primary functions shows a reciprocal influence, which may lead to curious observations and important deductions. Whether it will be in my power to obtain any further information in regard to them, is uncertain. If not, some one else can better accomplish the task.

Let me add that there is nothing unpleasant in the aspect of these boys. On the contrary, they must be viewed as presenting one of the most interesting objects of natural history, which have ever been known to scientific men.

You are at liberty to employ the above statement in such way as you think likely to be useful.

I have the honor to be yours, &c.

JOHN C. WARREN.

Wm. Sturgis, Esq. *Ed. Bos. Dai. Ad.*

24. *Extract from another account of the same.*—The precise effect of this physical union, on the intellectual faculties, the moral sentiments, and animal propensities of these boys,—its influence on the functions of the different organs, and how far it would communicate or modify the effects of the morbid or medicinal agents, are subjects on which we shall not enter. No opportunity has yet presented of observing the influence which disease or medicine in one, would exert on the other; but circumstances do not appear to justify the least suspicion of any mental individuality. Whispering in the ear of one, conveyed no sense of sound to the other Volatile salts applied to the nostrils of one, produced in the other only a curiosity to try the same experiment on himself. Pinching the arm of one, was attended by no sensation in the other. Being desirous of ascertaining if there was *any* point where both felt, we made an impression with the point of a pin in the exact vertical centre of their connecting link; both said it hurt them. We then made other impressions, extending them very gradually further from this point; the result was, that within the distance of three-fourths of an inch from the centre toward each boy, sensation was communicated to both by a single prick; beyond this it was excited in one only, the other perceiving it in no degree whatever. This experiment was remarkably satisfactory, and we apprehend that farther than here exhibited, the two youths must be considered, whilst in a state of health, as free and independent agents, and the functions of all the other organs as unconnected as those of their brains.

Twins generally resemble each other in intellect and disposition, as well as in person, and this is particularly the case with the boys in question. When to this natural resemblance we add the habit they have contracted of acting simultaneously and in concert, we shall be less surprised than we might at first be, at the facility with which their various movements are performed, and the quickness with which one responds to the inclinations of the other.

In the course of their voyage, they will not only run, we are told, and leap with great agility and without interfering with each other, but climb to the mast-head as fast as any sailor on board the ship. They are seldom observed to converse with each other, and the concert with which they act seems to be almost instinctive. In playing the game of drafts, e. g., which they learnt with great ease, being a people naturally fond of games and gambling, they were observed to decide on their moves almost instantaneously, and to make them with a quickness and air of decision sufficiently characteristick of all their movements. In the course of the game, sometimes the one and sometimes the other would make the move; they appeared to have the same plans, and always acquiesced in the moves of each other. Yet they sometimes *play against each other*; but so strong is their habit of co-acting, that such games go on with less freedom than when opposing a third person. Their alvine evacuations generally occur at the same time; their appetites and tastes are all very much alike; and they appear not only contented but happy, and extremely attached to each other. Capt. Coffin was informed by their mother, that she had borne seventeen children. Once she had three at a birth, and never less than two; though none of her other children were in any way deformed.

The question naturally arises in the mind of every observer, could not this connecting substance be divided without injury to the boys? —We do not pretend to solve this problem, which after all can only be decided by the experiment; but we hesitate not to say that, after several very accurate examinations, our impressions are that such a division would be a detriment only to the very respectable and obliging gentleman who offers them for exhibition. The anatomical structure of this bond of union is apparently simple, and we regard the fact that children so united should have been ushered into the world with safety to themselves and their mother, that they have escaped the ills and early fatality which usually attend such prodigies, that they should have grown up to the age of 18 years in the uniform exercise of mutual good will and a spirit of mutual accommodation, and that they should be so perfectly contented with their lot, and so happy in all the various unpleasant circumstances in which they are placed, as far more remarkable than that such a deformity should have existed. Instances of fœtuses united much more closly than are these boys, are by no means rare in the books or cabinets of anatomists. The mode of union is very various, being sometimes at the hips, backs or sides; several cases are related by Parey and Tulpius, in which the connection was at the abdomen. In the Philosophical Transactions is an account of two children thus united, born near Manchester, Eng. in 1752. 1748 Dr.

Parsons comunicated to the Royal Society an account of a still birth not very unlike the boys now exhibited. The fœtuses were united from the umbilicus to the upper part of the sternum, and the single chord by which they were nourished, entered the connecting medium at a central point on its lower surface. Dr. Cotton Mather communicated to a learned friend in England, a similar case of which he was eye-witness, and which occurred in this city in 1713; and a double fœtus, born in this county at a much later period, is now preserved and deposited, if we mistake not, in the anatomical cabinet of a neighbouring medical institution.

Most monsters have been still-born, and of the few who have been living, a very small proportion have survived many days. The most remarkable and as far as our memory goes, the only case on record of such monsters acquiring the adult age, occurred in Hungary more than a century ago. Two females Judith and Helen, born in Szona in 1701, were united at the lower part of the back. They had but one urethra and one passage for the fæcal evacuations. Their bodies, abating the deformed part alluded to, were well shaped and their faces beautiful. They were intelligent, and like the boys of Siam, not only contented, but in the language of their father, "both brisk and merry." Like them, also, these girls "had not their feeling common any where but in the place of their conjunction." When one stooped she lifted the other on her back, and whe one went forward the other was drawn backward. One would sometimes sleep whilst the other was awake, and though tenderly attached, their inclinations were not always the same. These Hungarian sisters were well educated and well bred; they could speak four different languages, and sing very prettily. They lived to the age of twenty-two years, during which time they were exhibited in different parts of Europe, and both died together in 1723.—*Bos. Med. Intel.*

25. *A case of twins united by the Umbilicus.* By Joshua Martin, M. D. of Xenia, Ohio.—On Monday the 31st of August 1829, I witnessed a most extraordinary *lusus naturæ* of which I am induced to publish a brief notice.

The wife of a gentleman of this vicinity was delivered on Saturday the 29th of August, at the close of the eighth month of utero-gestation, of two living children, of ordinary size, who were attached together by a round substance, of about three inches diameter, commencing at the ensiforme cartilage, or lower end of the breast bone, and extending down the abdomen.

The superior part of the attachment was hard and cartilaginous, formed by the ensiforme cartilage of the one extending across, and uniting with that of the other; below it was soft and gave the sensation to the touch, of a membranous sack, containing part of the abdominal viscera.

At the inferior part of the connecting medium, the skin was wanting; and at that point arose one umbilical chord which served both children.

Anastamosis, or union of the superficial veins of the two children could be distinctly perceived. They were both females and in every respect natural, except that one had two thumbs on the left hand. I saw them about forty-eight hours after their birth, one of them had then been dead twelve hours, and the other died in a few hours afterwards.—*Far. Rec. and Zenia Gaz.*

26. *General Phlebitis—or Purulent Concretions in Veins.*—The following case will prove a very valuable addition to the mass of facts brought forward by Mr. Arnott on phlebitis and its consequences.

*Case.* F. Gibeau, aged 28 years, was delivered of twins on the 8th July, 1826. On the 9th and 10th she went on well, though the lochia were scanty. On the 11th her breasts swelled and became painful, the pain extending to the axillæ, and along the left side of the chest, with rigors, fevers, some delirium, diarrhœa, and vomiting. Pains now seized various other articulations, and she was brought to the hospital on the 14th July. Her countenance was now pallid, features shrunk, tongue coated, dry, and red; thirst, abdomen tense and tender on pressure, a large umbilical hernia easily reducible, diarrhœa, vomiting, quick pulse, cough, oppression, knee-joints red, swelled, and painful—œdema of the lower extremties, which were tense, shining, white, elastic, and did not leave pits on pressure. The right mamma was swelled and shining. The diagnosis was —"active œdema of puerperal women"—"*œdeme actif des nouvelles accouchees:*" in other words, PHLEGMATIA DOLENS. Twenty ounces of blood were taken from the arm—lowest scale of regimen—emolient cataplasms to the breast and abdomen. The venesection was repeated the next two days, the blood being much inflamed. *The left arm became the seat of the great pain,* 19th July. There are two gangrenous spots on the right mamma—a similar appearance was presented on the labia pudendi. The left arm showed the same kind of œdematous swelling as the lower extremities. The phlegmasia, which was thus extended to so many organs and parts, was now treated by the tartrite of antimony, in doses of six grains per diem. This plan was pursued during six days—from the 20th to the 26th July, in the course of which time, the diarrhœa ceased; but the œdematous swellings remained. The diarrhœa returned on the 30th July, and continued till the patient's death, which took place on the 15th of August.

*Dissection.* We must pass over a great many minute particulars, in order to preserve the more important parts of the dissection. There were some points of hepatization in the lungs, but no purulent depots. The pharynx and œsophagus were completely lined with an exudation resembling soft morter, forming a tube that reached to the cardia, and there terminated. The mucous membrane of the œsophagus itself was pale when denuded. The peritoneum was healthy, as was the mucous membrane of the stomach and intestines; except some small portions of the colon. The uterus was the size of a fist, and its cavity filled with a sanguineo-purulent fluid. The parieties of the organ were livid—and, towards the fundus, in a state of molescence. There was a vast depot of suppuration about the left shoulder joint—but the other articulations, so swelled and inflamed during life, were now collapsed and colourless. The principal veins of the left arm were filled with a white tubular concretion, non-aderent to the internal surfaces of the vessels. The tubular concretion itself was filled with pus exactly similar to that found around the joint. The internal coats of the vein were remarkably pale. The iliac veins contained polypus concretions not at all analogous to those above described. The hypogastric and femoral veins, however, presented tubuler concretions filled with pus, down even to the ankle. Finally, the pulmonary *arteries* presented the same appearances as those described in the veins.

*Remarks.* When we consider the state of the internal surfaces of the vessels in which these concretions filled with pus, were found,—pale and non-adherent to the said concretions—we must conclude with the author that the phenomena cannot be legitimately traced to phlebitis. "I am rather disposed," says he, "to believe that this concretion was formed at the expense of the blood itself—and that it was the consequence of a mixture of pus, that is, an inorganic fluid, with one endowed with life." In whatever way the facts may be viewed, they tend to support the doctrine of the humoral pathology, so long and so unjustly ridiculed by sciolists and visionary solidists.—*Revue Medicale.—Med. Chir. Rev.*

27. *Varieties of Hydrocele—Modifications of the usual operations.*—The operation of injection for hydrocele is certainly, in the great majority of cases, extremely easy and safe in its performance, as well as successful in its results. Cases, however, occasionally occur, where untoward symptoms arise to thwart the objects of the surgeon, or even endanger the patient's existence. Hæmorrhage, either externally, or into the tunica vaginalis, is not very unfrequent, and tetanus has, in several instances, followed the operation. M. Dupuytren, who, from his situation of "chef des travaux anatomiques," has dissected a considerable number of hydroceles, believes that these accidents depend on a variation in the anatomical relations of the hydrocele, a variation of which the surgeon should be aware. The following case is adduced in illustration of the Baron's remarks:

*Case.* Auguste * * *, ætatis 13, of delicate constitution, was admitted into the Hotel Dieu, with a hydrocele of six months' standing in the left side. The whole circumference of the hydrocele might be grasped without pain, but the instant the upper part was touched, the patient experienced the sensation of the testicle having been meddled with. On using the lighted taper, the testicle was found to be lodged in this part; and, "from the extremity of one of its diameters," the cord passed in front of the scrotum to gain the inguinal canal. In short, the testicle occupied the summit of the hydrocele, and the spermatic cord was situated along its anterior part. Under these circumstances, M. Dupuytren performed the operation at the middle and external part of the scrotum, in order to avoid the lesion of the organs alluded to above.

M. Dupuytren believes that, had the usual operation been resorted to here, and the testicle and cord been wounded, as they must, it is not improbable that serious mischief might have followed. At all events, he recommends, and we think very justly, that prior to thrusting in the trocar, the position of the testicle and spermatic cord should be carefully ascertained. *Ibid.*

28. *On the Reduction of Herniæ.* By M. Dupuytren.—The means of reducing hernia are various, and more or less efficient. The application on the hand, or taxis, is the most methodic and advantageous; it is modified according to the kind of hernia, its size, and other circumstances. Among the empirical methods adopted for the same purpose, there is one which approaches to this. The patient being laid upon his back, the feet are raised as high as possible, leaving the head and shoulders upon the ground; the weight of the viscera in the abdomen acts upon the portion of bowel in the hernia, dragging it towards the interior, sometimes effecting the reduction. Here there is a mechanical action, not from without, inwards, as in the taxis, but in an opposite direction. Various topical applications are made to strangulated herniæ; some of these, as cold water, &c. are intended to diminish the volume of the parts in the sac. The action of cold produces several effects; it increases the tone of muscular parts, often, indeed, giving rise to sudden contractions, capable of overcoming the obstacle which has been opposed to the passage of the intestinal contents; so that strangulated herniæ are sometimes speedily reduced by the affusion of very cold water. Ice, applied with perseverance, condenses the liquids and gas, and thus also facilitates their return into the abdomen. Other local applications appear intended to act upon the secretion of the mucous membrane of the part; such as cataplasms of senna, gratiola, &c. These are asserted to have good effects in the cases of elderly persons, in whom it is well known that the slowness of the peristaltic motion more frequently occasions overloading than actual strangulation; a circumstance which must be kept in mind, that we may not trust too much to these measures where the patient is young, and the case one of genuine incarceration. As experience has demonstrated that the manner in which purgatives act is by facilitating the expulsion of the contents of the herniæ, it may be asked why more active substances are not employed; for example, the croton oil?

The nausea and vomiting so frequently present in such cases, does not always contra-indicate the use of purgatives, as they sometimes succeed when more methodical treatment has failed. With regard to enemata, they ought never to be omitted unless there be evident symptoms of inflammation.

Some local applications are intended to produce relaxation at the point of strangulation; thus the extract of belladonna has recently appeared to produce good effects in the hands of M. St. Amand.

The rapid sinking of the patient frequently produces the relaxation favorable to the reduction of the herniæ. This is effected by means of copious general bleeding; the application of leeches to the tumour; and long-continued immersion in a warm bath; means which in general are not employed with sufficient energy. These observations show, that the operation is not the only resource in such cases; but it is a very important point that the operation be not delayed after its necessity has become obvious. A consideration of all the circumstances can alone lead to a satisfactory conclusion. The following case will show that at the Hotel Dieu other means are tried before operating:

"A woman, aged fifty, had laboured under crural hermit of the right side for more than ten years. It frequently became obstructed, but repose, the horizontal posture, and the taxis, had always sufficed to restore it. On the 24th of February, after some efforts to carry a load, the tumour suddenly became the size of a small egg, and symptoms of strangulation manifested themselves with violence. The usual methods to produce reduction were adopted without avail. Next day she was brought to the hospital, when she was bled from the arm, the tumour covered with leeches, and she was put into a warm bath, where she remained nearly two hours. At the end of this time faintness came on, when the 'interne' took the opportunity of applying the taxis, by which means he entirely reduced the herniæ.

M. Dupuytren stated, that at the Hotel Dieu they succeeded in reducing only one-third of the herniæ brought to them, while in civil life at least two-thirds were reduced. Among the causes of this difference, the principal is, that those cases in which reduction has already been attempted without success are generally sent to the hospital. In the better ranks of life the causes of strangulation are much less frequent; the patients much more ready to call in assistance. If we are in haste to operate on such individuals, some of those who die might possibly be saved; but at the same time we run the risk of practising an operation which is not called for. Besides, there is never more than twelve hours between each visit; and it is generally in this interval that reduction takes place. Prudence, therefore, requires that we ought not to be in too great haste; and experience proves that gangrene of the intestine neither comes on so easily nor so soon as is generally said. We are now less imposed upon by the brown colour which the organ assumes in consequence of the constriction; and knuckles of intestine are now replaced in the abdomen, the appearance of which would formerly have been regarded as indicating the necessity of establishing an artificial anus. It is necessary, then, to multiply the means of reduction; to persevere as long as possible in their employment, and only to despair of their success when the continuance of the local mischief, and the increase of the general symptoms, give rise to well-grounded apprehensions.—*Clinical Lectures of M. Dupuytren, La Clinique. Ibid.*

28. *Supposed Artificial Diamonds.*—M. Thenard gave an account of the experiments made by himself, MM. Dumas and Cagniard de la Tour, to verify the trials by which the latter thought he had obtained the power of crystalizing carbon, and forming diamond. An accurate analysis of these crystals, which had no colour, proved however, that they were only silicates, and not artificial diamond.—*Annales de Chimie*, 1829.—*Amer. Jour. of the Med. Sciences.*

